# Postchallenge responses of nitrotyrosine and TNF-alpha during 75-g oral glucose tolerance test are associated with the presence of coronary artery diseases in patients with prediabetes

**DOI:** 10.1186/1475-2840-11-21

**Published:** 2012-03-07

**Authors:** Chih-Sheng Chu, Kun-Tai Lee, Kai-Hong Cheng, Min-Yi Lee, Hsuan-Fu Kuo, Tsung-Hsien Lin, Ho-Ming Su, Wen-Chol Voon, Sheng-Hsiung Sheu, Wen-Ter Lai

**Affiliations:** 1 Division of Cardiology, Department of Internal Medicine, Kaohsiung Medical University Hospital, Kaohsiung, Taiwan; 2 Department of Internal Medicine, Faculty of Medicine, College of Medicine, Kaohsiung, Taiwan; 3 Department of Internal Medicine, Kaohsiung Municipal Ta-Tung Hospital, Kaohsiung, Taiwan; 4 Graduate Institute of Medicine, Kaohsiung Medical University, Kaohsiung, Taiwan

**Keywords:** Postchallenge hyperglycemia, Inflammation, Oxidative stress, Nitrotyrosine oral, Glucose tolerance test, Coronary artery disease

## Abstract

**Background:**

Meta-analysis has demonstrated an exponential relationship between 2-hr postchallenge hyperglycemia and coronary artery disease (CAD). Pulsatile hyperglycemia can acutely increase proinflammatory cytokines by oxidative stress. We hypothesized that postchallenge proinflammatory and nitrosative responses after 75 g oral glucose tolerance tests (75 g-OGTT) might be associated with CAD in patients without previously recognized type 2 diabetes mellitus (T2DM).

**Methods:**

Serial changes of plasma glucose (PG), tumor necrosis factor-alpha (TNF-α), interleukin-6 (IL-6) and nitrotyrosine levels were analyzed during 75 g-OGTT in 120 patients (81 male; age 62 ± 11 years) before coronary angiography. Patients were classified as normal (NGT; 42%), impaired (IGT; 34%) and diabetic (T2DM; 24%) glucose tolerance by 75 g-OGTT.

**Results:**

Postchallenge hyperglycemia elicited TNF-α, IL-6 and nitrotyrosine levels time-dependently, and 2-hr median levels of TNF-α (7.1 versus 6.4 pg/ml; *P *< 0.05) and nitrotyrosine (1.01 versus 0.83 *μ*mol/l; *P *< 0.05), but not IL-6 or PG, were significantly higher in patients with CAD in either IGT or T2DM groups. After adjusting risk factors and glucose tolerance status, 2-hr nitrotyrosine in highest quartiles (OR: 3.1, *P *< 0.05) remained an independent predictor of CAD by logistic regression analysis.

**Conclusions:**

These results highlight postchallenge proinflammatory and nitrosative responses by 75 g-OGTT, rather than hyperglycemia *per se*, are associated with CAD in patients without previous recognized diabetes.

## Background

Type 2 diabetes mellitus (T2DM) is widely accepted as an independent risk factor of atherosclerotic cardiovascular diseases [1]. Meta-analysis has demonstrated an exponential relationship between 2-hr postchallenge hyperglycemia and cardiovascular risks, and this relationship might extend below the diabetic threshold [2]. Epidemiological studies have also shown that subjects with impaired glucose tolerance (IGT) have a raised risk of cardiovascular morbidity and mortality [3-6].

Chronic diabetic macrovascular complications might be attributed to the exaggerated postprandial glycemic excursion, rather than chronic fasting hyperglycemia [7]. Glycemic excursion promotes atherogenesis by several possible mechanisms, including oxidative stress, endothelial dysfunction, reduced nitric oxide bioavailability, impaired vasodilation and formation of advanced glycated end-products [8]. Postprandial "hyperglycemic spike" is suggested to have impact on both endothelial function and oxidative stress, and consequently, the development of cardiovascular disease. This phenomenon is not only evident in patients with T2DM but also extended into subjects with IGT [3,4]. However, the question remains whether hyperglycemia is the marker or the cause of atherosclerotic changes in patients without overt T2DM [9,10].

Inflammation and oxidative stress elicited by either hyperglycemia or hyperlipidemia play an interactive role in the pathogenesis of insulin resistance, diabetes and atherosclerosis [11-13]. Hyperglycemia spike had been shown to acutely increase the levels of circulating proinflammatory cytokines, including tumor necrosis factor-alpha (TNF-α) and interleukin-6 (IL-6), in subjects with IGT and these responses are attenuated by antioxidant [14]. In addition, pulsatile hyperglycemia increases these cytokines to a greater extent than continuous hyperglycemia during similar glycemic levels [4,14]. Nitrotyrosine has been indirectly inferred as a marker of postprandial oxidative stress because of the production of peroxynitrite by acute hyperglycemia [14,15]. Experimental study has revealed the presence of nitrotyrosine from extensive nitration of protein tyrosine in human coronary atherosclerotic lesions [16]. Clinically, correlations between postprandial hyperglycemia and the generation of nitrotyrosine have been demonstrated in patients with T2DM or gestational diabetic pregnancies [16,17]. Moreover, elevated baseline nitrotyrosine levels have recently been shown in association with coronary artery disease (CAD) and modulated by statin therapy [18]. However, it remains unknown whether the dynamic changes of circulating TNF-α, IL-6 and nitrotyrosine induced by postchallenge hyperglycemia might be associated with CAD in patients without previously recognized T2DM.

We sought to investigate the dynamic responses of circulating proinflammatory cytokines (TNF-α and IL-6) and nitrosative stress (nitrotyrosine) induced by postchallenge hyperglycemia after 75 g oral glucose tolerance test (75 g-OGTT) and to evaluate their associations with the presence of CAD in patients without previous diagnosed T2DM.

## Methods

### Patients enrollment

A total of 134 consecutive patients who underwent both coronary arteriography and 75 g-OGTT test at Kaohsiung Medical University Hospital between April 2005 and May 2006 were enrolled. Seven patients were excluded because of being treated for T2DM, having fasting plasma glucose (PG) concentration ≧ 126 mg/dl (7.0 mmol/l) or hemoglobin A_1C _≧ 6.5% [19]. Another 7 patients with valvular heart disease, hypertrophic or dilated cardiomyopathy, chronic renal insufficiency (serum creatine > 2.0 mg/dl) or acute coronary events within 6 months were also excluded. Thus, 120 patients without previously overt T2DM were investigated prospectively in this study. A standard questionnaire was applied to all patients to acquire their personal medical histories, traditional cardiovascular risk factors, symptoms of angina pectoris and current medication. All patients underwent coronary arteriography because of effort angina pectoris, positive exercise treadmill test or positive stress myocardial perfusion scintigraphy. This study complies with the Declaration of Helsinki, and the study protocol was approved by the Institutional Review Board at Kaohsiung Medical University and written informed consents were obtained from all patients

### 75 g-OGTT and blood sampling

After a 12-hr overnight fast during hospital stay, patients were asked to stay in a comfortable supine position with a room temperature between 20° and 24°C. Intravenous lines were inserted into a large antecubital vein of one arm for blood sampling between 8 a.m. and 10 a.m. and patency was preserved by a slow saline infusion (0.9% NaCl).

After the ingestion of a 75 g oral glucose, serial venous blood samples (10 ml) for PG were collected before (PG-0) and at 30 (PG-30), 60 (PG-60), 90 (PG-90) and 120 (PG-120) minutes, and also for TNF-α, IL-6 and nitrotyrosine at 0, 60 and 120 minutes. Blood samples were collected into pyrogen-free vacuum collection tubes without additives for measurements of serum TNF-α, IL-6, lipid profiles and hemoglobin A_1C_; or with EDTA as anticoagulant for the measurement of PG and nitrotyrosine. The blood collection tubes were immediately immersed in melting ice and centrifuged at 1500 *g *for 10 min within 20 min (plasma) or allowed to clot before centrifugation (serum). All samples were frozen and stored at -80°C until analysis.

### Biochemical measurements

PG concentrations were measured by the standard glucose oxidase method. Total cholesterol and triglycerides were determined by an enzymatic method. High-density lipoprotein cholesterol (HDL-C) was measured after phosphotungstic acid/MgCl2 precipitation on fresh plasma. Low-density lipoprotein cholesterol (LDL-C) was calculated using the Friedewald formula. The hemoglobin A_1C _and fasting PG levels were analyzed by the hospital central laboratory, with a normal hemoglobin A_1C _reference range of 4.5 ~ 6.0%.

### Enzyme immunoassays of TNF-α, IL-6 and nitrotyrosine

Serum levels of both TNF-α, IL-6 were measured in duplicate by ELISA kits (R & D systems Inc., Minneapolis, MN, USA) [14]. The concentrations of plasma nitrotyrosine were determined in duplicate by ELISA kits (Northwest, Life Science Specialties, Vancouver, WA, Canada) [17]. Dilution curves of analyzed samples were parallel to those of standard. Intra-assay and interassay coefficients of variation were 3.7% and 5.9%, respectively for TNF-α; 3.8% and 5.6% respectively for IL-6; and 3.3% and 5.5% respectively for nitrotyrosine.

### Coronary arteriography

Coronary arteriography followed after the 75 g-OGTT was completed in each patient that same morning. Patients were diagnosed to have CAD if coronary arteriography revealed any stenosed vessel with > 50% luminal narrowing by quantitative coronary analysis in the main coronary arteries or major branches, otherwise were non-CAD [20]. The numbers of stenosed or occluded vessels were also calculated to define the clinical (1- to 3-vessels disease) CAD score. The interpretations of all coronary arteriograms were done by three observers who were blinded to the clinical and laboratory data. The interclass correlation coefficient for intrarater reliability was 1.1 for the clinical CAD scores.

### Glucose tolerance status

All patients were categorized by the final results of 75 g-OGTT according to the diagnostic thresholds of the Expert Committee on the Diagnosis and Classification of Diabetes Mellitus [19]. Firstly, patients with fasting PG < 100 mg/dl (5.6 mmol/l) and 100-125 mg/dl (5.6-6.9 mmol/l) were considered to have normal and impaired fasting glucose, respectively. Secondly, patients with PG at 120 minutes < 140 mg/dl (7.8 mmol/l), 140-199 mg/dl (7.8-11.0 mmol/l), and ≧ 200 mg/dl (11.1 mmol/l) were considered to have normal (NGT), impaired (IGT), and diabetic GT (T2DM), respectively. Patients with IGT or T2DM were considered to have abnormal GT (AbnGT). Both status of GT and CAD were taken into consideration for further subgroup analysis.

### Statistical analysis

Continuous variables were presented as mean (± SD) where applicable. Levels of TNF-α, IL6 (both in pg/ml) and nitrotyrosine (in *μ*mol/l) were not normally distributed and values were presented as median and interquartile range. Categorical variables were compared by chi-square analyses. Differences among groups were examined by Kruskal-Wallis test followed by Mann-Whitney *U *test with Bonferroni correction. Associations were determined using the Pearson and Spearman correlation coefficients for continuous measurements. Logistic regression models were used to calculate odds ratio (ORs) associated with the second, third, and the highest quartile of TNF-α, IL-6 and nitrotyrosine, respectively, and compared with the corresponding lowest quartile for the presence of CAD. Trends were assessed with Cochran-Armitage tests. All *p *values were two-tailed and all confidence intervals were computed at the 95% level. *P *value < 0.05 was considered statistically significant. Statistical analysis was performed using SPSS software version 11.0 (SPSS Inc., Chicago, IL, USA).

## Results

### Patient characteristics

Baseline characteristics of patients categorized according to the presence of CAD were summarized in Table 1. In this study, patients with CAD were significantly older and more likely to be male. No significant differences were demonstrated between the CAD and non-CAD groups with regards to systolic or diastolic blood pressure, body mass index or waist-tohip ratio. No significant differences in hemoglobin A_1C_, fasting glucose, lipid profiles or current medications can be shown between the CAD and non-CAD groups in our study. In terms of fasting glucose status, there were 55 (46%) patients had normal fasting glucose and 65 (54%) patients with impaired fasting glucose at baseline. However, no significant difference was found in the percentage of impaired fasting glucose between the CAD and non-CAD patients.

**Table 1:** Baseline characteristics of patients categorized according to the presence of CAD (n = 120)

Characteristic	CAD (n = 65)	Non-CAD (n = 55)	*P values*
Age (yrs)	64.9 ± 10.6*	59.1 ± 9.6	< 0.05
Sex (F/M)	13/52*	26/29	< 0.05
SBP (mmHg)	132 ± 22	129 ± 19	0.238
DBP (mmHg)	81 ± 14	77 ± 12	0.163
Body mass index (kg/m^2^)	26.6 ± 3.3	25.9 ± 4.8	0.835
Waist-to-hip ratio	0.85 ± 0.10	0.83 ± 0.15	0.729
Fasting plasma glucose (mg/dl)	103.3 ± 12.3	101.7 ± 13.3	0.527
Impaired fasting glucose [n (%)]	35 (53.9%)	30 (54.5%)	0.938
HbA_1C _(%)	5.4 ± 0.9	5.2 ± 0.7	0.725
Family history of CAD [n (%)]	12 (18.4%)	8 (14.5%)	0.815
Past history [n (%)]			
Hypercholesterolemia	34 (52.3)	28 (51.0)	0.078
Hypertension	47 (72.3)	37 (67.2)	0.159
Alcohol intake	22 (33.8)	22 (40.0)	0.875
Current smoker	18 (27.7)	14 (25.4)	0.753
Lipid profiles (mg/dL)			
Total cholesterol	215 ± 48	208 ± 41	0.238
LDL cholesterol	127 ± 37	119 ± 42	0.105
HDL cholesterol	41 ± 10	43 ± 12	0.369
Triglycerides	217 ± 126	209 ± 138	0.245
Concomitant medication [n (%)]			
Aspirin/Clopidogrel	37 (56.9)	25 (45.4)	0.095
β Adrenergic blockers	18 (27.7)	16 (29.1)	0.358
Calcium channel blockers	44 (67.7)	36 (65.4)	0.259
ACE inhibitor/ARB	29 (44.6)	23 (41.8)	0.347
Diuretics	24 (36.9)	18 (32.7)	0.135
Statin	33 (50.8)	26 (47.2)	0.085
Fibrate	5 (7.7)	4 (7.3)	0.468

*P < 0.05; ACE = angiotensin-converting enzyme; ARB: angiotensin II receptor blockers; BMI: body mass index; HbA_1C_: hemoglobin A_1C_; HDL: high density lipoprotein; LDL: low density lipoprotein

### Glucose tolerance status and CAD

**Figure 1:**
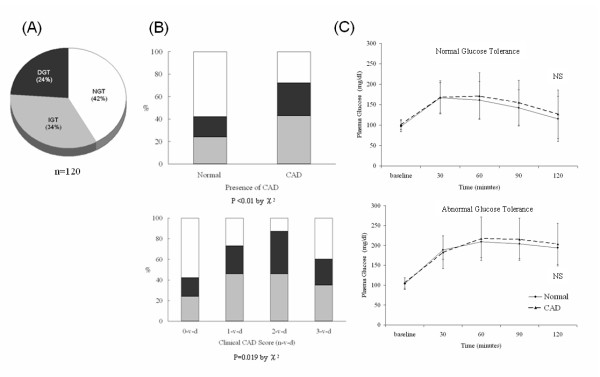
**The distributions of glucose tolerance (GT) and CAD status, as well as the PG response curve during 75 g-OGTT. (A) After 75 g OGTT, the proportions of IGT and T2DM are 34% and 24%, respectively**. (**B**) Frequency distribution of GT status in the presence of CAD or among the clinical (1- to 3- vessels diseases) scores. Significantly higher percentages of AbnGT (IGT and/or T2DM) are associated with CAD by chi-square analysis (both *P *values < 0.05). The white, light grey and dark grey areas indicate the percentage of NGT, IGT and T2DM, respectively. (**C**) No significant differences in PG response curves between CAD and non-CAD patients in either NGT or AbnGT group during the entire course of 75 g-OGTT.

The proportions of patients with IGT (n = 41, 34%) and T2DM (n = 29, 24%) documented after 75-g OGTT test were relatively high, and less than half of our study subjects had NGT (n = 50, 42%) (Figure 1A). Moreover, subcategory analysis by fasting glucose status, there were 47% had NGT, 40% had IGT, and 13% had T2DM in patient with normal fasting glucose (n = 55), and 39%, 29% and 34%, correspondingly, in patient with impaired fasting glucose (n = 65).

The frequency distributions of NGT, IGT and T2DM among patients grouped by the presence of CAD and number of stenosed coronary arteries were illustrated (Figure 1B). The chi-square analyses revealed that patients with IGT and T2DM were significantly associated with the presence of CAD and the number of diseased coronary arteries involved compared to patients with NGT (both *P *values < 0.05). However, serial PG levels during the entire course of 75 g-OGTT in patients with NGT or AbnGT (IGT and T2DM) did not show any significant difference in terms of the presence or absence of CAD (Figure 1C). The time to peak PG values tended to be later (at 60-90 minutes) in patients with AbnGT than with NGT (at 30-60 minutes).

### Responses of proinflammatory cytokines and nitrotyrosine induced by postchallenge hyperglycemia

**Figure 2:**
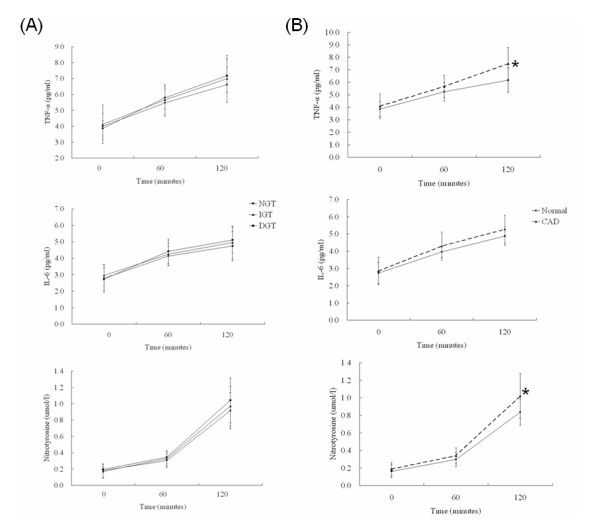
**Postchallenge TNF-α, IL-6 and nitrotyrosine responses in (A) patients with different GT status or (B) patients with or without CAD, after 75 g-OGTT**. Circulating levels of TNF-α, IL-6 and nitrotyrosine are consistently and time-dependently elevated within 2-hr after 75 g-OGTT. No significant differences of these cytokines levels among NGT, IGT or T2DM status are demonstrated. However, significant increases of TNF-α and nitrotyrosine, but not IL-6, could be demonstrated at 120 minutes after 75 g-OGTT in CAD patients in comparison with non-CAD patients. *P < 0.05.

Serial changes of TNF-α, IL-6 and nitrotyrosine levels after 75 g-OGTT at 0, 60 and 120 minutes among patients with NGT, IGT, and T2DM (Figure 2A) and between CAD and non-CAD patients (Figure 2B) were illustrated. At baseline, there were no significant differences in values of TNF-α, IL-6 or nitrotyrosine among 3 different glucose tolerance statuses or between CAD and non-CAD groups. Circulating levels of TNF-α, IL-6 and nitrotyrosine were consistently and time-dependently elevated after postchallenge hyperglycemia. No significant differences of postchallenge TNF-α, IL-6 and nitrotyrosine levels among patients with different GT status could be shown. However, significant differences of both TNF-α (7.1 [6.7-8.3]) versus 6.4 [5.8-7.9] pg/ml) and nitrotyrosine (1.01 [0.82-1.17] versus 0.83 [0.74-1.01] *μ*mol/l) were able to be demonstrated at 2-hr after 75 g-OGTT between CAD and non-CAD patients (both *P *values < 0.05) (Figure 2 and Table 2). The differences of IL-6 (5.2 [4.2-5.8] versus 4.8 [4.3-5.0] pg/ml) levels at 2-hr after 75 g-OGTT load did not achieve significance between CAD and non-CAD patients. However, for patients with coronary 3-vessel diseases, significantly higher levels of 2-hr IL-6 (5.4 [4.8-5.8] versus 4.8 [4.3-5.0] pg/ml, *p *< 0.05) could be demonstrated in comparison with patients without CAD (Table 2).

**Table 2:** Comparisons of plasma glucose, TNF-α, IL-6 and nitrotyrosine after 75 g OGTT at 120 minutes by the presence of coronary artery disease and the number of significantly stenosed coronary arteries

	Subgroup	n	PG-120 (mg/dl)	*p*	TNF-α (120) (pg/ml)	*p*	Post- hoc	IL-6 (120) (pg/ml)	*p*	Post- hoc	NT (120) (*μ*mo/l)	*p*	Post-hoc
CAD	(-)	55	155 ± 60	0.063	6.4(5.8-7.9)	< 0.05		4.8(4.3-5.0)	0.121		0.83(0.74-1.01)	< 0.05	
	(+)	65	177 ± 56		7.1(6.7-8.3)			5.2(4.6-5.8)			1.01(0.82-1.17)		
n-V-D	(a) 0-v-d	55	155 ± 61	0.091	6.4(5.8-7.9)	< 0.05	b > a	4.8(4.3-5.0)	< 0.05	d > a	0.83(0.74-1.01)	< 0.05	b > a
	(b) 1-v-d	30	170 ± 46		7.3(5.7-7.8)		c > a	5.2(4.4-5.5)			0.92(0.89-1.15)		c > a
	(c) 2-v-d	15	187 ± 59		6.9(6.3-7.8)		d > a	5.1(4.5-5.4)			0.96(0.79-1.18)		d > a
	(d) 3-v-d	20	172 ± 57		7.4(6.6-8.8)			5.4(4.8-5.8)			0.99(0.82-1.25)		

* By pair-*t *test or ANOVA with significant pairs from Turkey's pairwise comparison. IL6: interleukin-6; NT: nitrotyrosine; PG: plasma glucose; TNF-α: tumor necrosis factor-alpha; OGTT: oral glucose tolerance test

**Figure 3:**
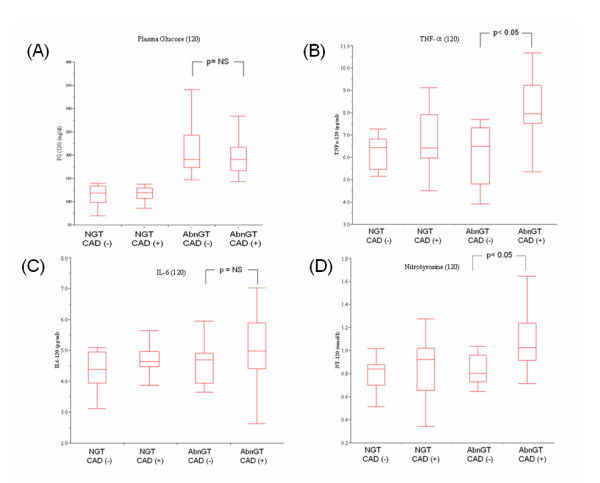
**Box and whisker plots of PG, TNF-α, IL-6 and nitrotyrosine levels at 2-hr after 75 g-OGTT**. Each plot indicates the median (middle bar in rectangle), 10^th ^(bottom of bar), 25^th ^(bottom of rectangle), 75^th ^(top of rectangle), and 90^th ^centiles (top of bar) in patients categorized by the presence of CAD and GT status. Patients with CAD and AbnGT have significantly higher postchallenge TNF-α and nitrotyrosine levels at 2-hr compared to those without CAD, whereas IL-6 and glucose concentrations are not significantly different either in patients with NGT or AbnGT. *p < 0.05 by Kruskal-Wallis test followed by Mann- Whitney *U *test with Bonferroni correction.

The values of PG, TNF-α, IL-6 and nitrotyrosine categorized simultaneously with GT status and the presence of CAD were summarized in Table 3. All the PG values ranging from 0 to 120 minutes were not significantly different between CAD and non-CAD patients in each GT group separately. However, postchallenge TNF-α and nitrotyrosine levels at 2-hr were both significantly higher in either IGT or T2DM patients with CAD compared to those without. Furthermore, when the status of IGT plus T2DM were taken together into consideration as AbnGT, both postchallenge TNF-α (7.8 [7.4-9.0] versus 6.4 [4.5-7.3] pg/ml) and nitrotyrosine (1.02 [0.92-1.25] versus 0.81 [0.74-0.96] *μ*mol/l) levels at 2-hr were still able to be demonstrated in AbnGT patients with or without CAD (both *P *values < 0.05) (Figure 3). However, no significant differences of PG-120 or IL-6 at 2-hr could be found in CAD or non-CAD patients, in either NGT or AbnGT groups. It was also noteworthy that no such significant differences of postchallenge TNF-α and nitrotyrosine at 2-hr could be demonstrated in NGT patients with or without CAD.

**Table 3:** Comparison of proinflammatory cytokines and oxidative markers before and after 75 g oral glucose tolerance test (75 g-OGTT) in patients categorized according to glucose tolerance status and the presence of CAD

	NGT (n = 50)		IGT (n = 41)		T2DM (n = 29)	
	CAD (n = 18)	Non-CAD (n = 32)	CAD (n = 28)	Non-CAD (n = 13)	CAD (n = 19)	Non-CAD (n = 10)
PG (mg/dl)						
PG-0	99 ± 11	98 ± 12	102 ± 11	100 ± 13	112 ± 13	108 ± 17
PG-30	163 ± 36	173 ± 43	175 ± 31	182 ± 13	203 ± 47	188 ± 34
PG-60	165 ± 44	165 ± 42	196 ± 38	204 ± 42	248 ± 60	216 ± 40
PG-90	152 ± 27	138 ± 28	187 ± 31	186 ± 21	257 ± 52	225 ± 43
PG-120	118 ± 15	112 ± 23	172 ± 17	170 ± 14	250 ± 51	246 ± 29
PG-150	114 ± 29	88 ± 23	148 ± 33	139 ± 19	204 ± 65	164 ± 57
PG-180	83 ± 26	85 ± 19	127 ± 40	104 ± 31	172 ± 69	167 ± 56
TNF-α (pg/ml)						
TNF-α (0)	3.8 (3.4-4.9)	3.8 (3.5-4.0)	3.9 (3.6-4.9)	3.6 (3.0-4.2)	3.8 (3.6-4.0)	3.9 (3.4-4.3)
TNF-α (60)	5.7 (5.1-5.9)	5.3 (5.0-5.8)	5.7 (5.4-6.1)	5.3 (5.2-5.9)	5.8 (5.5-5.9)	5.4 (5.3-6.7)
TNF-α (120)	6.4 (5.9-7.9)	6.5 (5.5-6.8)	7.5* (6.8-8.3)	6.4 (3.5-7.1)	7.6† (6.5-7.8)	6.2 (5.8-8.1)
IL-6 (pg/ml)						
IL-6 (0)	2.5 (2.2-3.4)	2.5(2.3-3.1)	3.2 (2.4-3.4)	3.0 (2.3-3.5)	2.5(2.0-3.4)	3.2(2.6-3.4)
IL-6 (60)	4.2(3.7-4.9)	4.0(3.9-4.2)	4.4 (3.7-5.0)	4.3 (3.7-4.6)	4.5(3.7-5.2)	4.3(3.8-4.6)
IL-6 (120)	4.7(4.4-5.0)	4.4(3.9-4.9)	5.0 (4.4-7.2)	4.4 (3.5-5.4)	5.2(3.0-5.8)	4.8(4.3-5.0)
NT (*μ*mol/l)						
NT (0)	0.23 (0.12-0.24)	0.17 (0.15-0.23)	0.17 (0.14-0.23)	0.18 (0.04-0.25)	0.20 (0.18-0.23)	0.17 (0.14-0.18)
NT (60)	0.34 (0.21-0.36)	0.33 (0.25-0.35)	0.33 (0.28-0.38)	0.35 (0.16-0.36)	0.35 (0.31-0.39)	0.29 (0.26-0.37)
NT (120)	0.92 (0.65-1.02)	0.84 (0.72-0.87)	0.98* (0.80-1.19)	0.88 (0.76-0.98)	1.02† (0.85-1.27)	0.87 (0.79-1.16)

Data are displayed in mean ± SD or median (interquartile range); *p < 0.05 represents the significant differences between normal and CAD in IGT group; †*P *< 0.05 represents the significant differences between normal and CAD in T2DM group; IL-6: interleukin-6; NT: nitrotyrosine; PG: plasma glucose; TNF-α: tumor necrosis factor-alpha

### Correlations of proinflammatory and nitrosative cytokines and postchallenge hyperglycemia

Baseline TNF-α, IL-6 and nitrotyrosine levels did not significantly correlate with baseline PG in this study. However, after 75-g glucose challenge, significant correlations of PG-120 concentrations were demonstrated with the 2-hr levels of TNF-α (r = 0.195, *P *< 0.05) and nitrotyrosine (r = 0.352, *P *< 0.05), but not IL-6. There were no significant correlations of postchallenge TNF-α, IL-6 or nitrotyrosine levels at 2-hr with age, fasting glucose, hemoglobin A_1C_, traditional cardiovascular risk factors and lipid profiles.

### Odds ratios of CAD risk by quartiles of postchallenge proinflammatory and nitrosative cytokines

The crude and adjusted odds ratios of CAD risk according to quartiles of postchallenge TNF-α, IL-6 and nitrotyrosine, respectively, at 120 minutes after 75 g-OGTT by logistic regression analysis are shown in Table 4. Unadjusted TNF-α (120) level in 4^th ^quartile was significantly associated with CAD (OR: 2.5, 95% CI, 1.3-6.9; *P *< 0.05). Unadjusted nitrotyrosine (120) levels in 3^rd ^(OR: 2.7, 95% CI, 1.7-4.5) and 4^th ^quartiles (OR: 4.9, 95% CI, 2.7-8.6) were both significantly predictors of CAD (both *P *values < 0.05). After adjustments for age, sex, body mass index, blood pressure, hemoglobin A_1C_, lipid profiles and GT status, nitrotyrosine (120) levels in 4^th ^quartiles (OR: 3.1, 95% CI, 2.2-5.3; *P *< 0.05) remained as a powerful predictor of CAD.

**Table 4:** Odd ratios of CAD to quartiles of TNF-α, IL-6 and nitrotyrosine after 75 g OGTT at 120 minutes

			Quartiles		
					*P *value‡
Characteristics	1	2	3	4	
**TNF-α (120) (pg/ml)**	**< 6.0**	**6.0-6.8**	**6.8-7.6**	**> 7.6**	

No. of CAD	13	16	15	21	
No. of Non-CAD	15	20	9	11	
Crude OR	1.0	1.3 (0.7-2.3)	1.5 (0.8-4.5)	2.5 (1.3-6.9)*	< 0.05
Adjusted OR†	1.0	1.2 (0.5-2.1)	1.4 (0.7-3.1)	1.9 (0.9-3.3)	0.053
			Quartiles		
					*P *value‡
	1	2	3	4	

IL-6 (120) (pg/ml)	< 4.0	4.0-5.0	5.0-5.5	> 5.5	

No. Of CAD	14	15	13	23	
No. of Non-CAD	18	22	10	5	
Crude OR	1.0	1.1 (0.9-2.6)	1.5 (0.7-3.5)	1.9 (0.9-4.2)	0.078
Adjusted OR†	1.0	1.2 (0.8-2.1)	1.3 (0.8-2.9)	1.5 (0.7-3.9)	0.121
			Quartiles		
					*P *value‡
	1	2	3	4	

NT (120) (*μ*mo/l)	< 0.70	0.70-0.90	0.91-1.10	≧ 1.10	

No. Of CAD	4	22	17	22	
No. of Non-CAD	7	23	16	9	
Crude OR	1.0	1.5 (0.9-2.6)	2.7 (1.7-4.5)*	4.9 (2.7-8.6)*	< 0.01
Adjusted OR†	1.0	1.2 (0.7-2.1)	1.7 (0.8-3.6)	3.1 (2.2-5.3)*	< 0.05

*P values < 0.05. Crude odd ratios (ORs) and confidence intervals (CIs) were calculated using logistic regression models comparing risk of each of the upper 3 quartiles for a given parameter to the lowest quartile. † Adjusted for age, gender, body mass index, smoking, lipid profiles, medication and glucose tolerance status by conditional logistic regression analysis. ‡*P *values for trend across quartiles

## Discussion

The major findings in this study were as follows: firstly, postchallenge hyperglycemia can rapidly elicit circulating TNF-α, IL-6 and nitrotyrosine levels after 75 g-OGTT in all patients regardless of glucose tolerance status or the presence or absence of CAD. Secondly, although patients with AbnGT were more likely to have CAD, there were no significant differences of postchallenge hyperglycemia ranging from PG-0 to PG-120 between CAD and non-CAD patients in either NGT or AbnGT groups. Thirdly, postchallenge TNF-α and nitrotyrosine levels at 2-hr, rather than hyperglycemia *per se*, were associated with the presence of CAD. Finally, after adjusting traditional cardiovascular risk factors and glucose tolerance status, nitrotyrosine at 2-hr after 75 g-OGTT in highest quartiles remained a powerful predictor of CAD.

### High prevalence of AbnGT in patients referred for coronary arteriography

Our study results revealed that, in patients referred for coronary arteriography with previously unrecognized T2DM, in which only 42% of patients had NGT and 24% had newly detected T2DM. This finding was consistent with previous reports that higher prevalence of AbnGT, ranging from 50% ~ 60%, can be found in patients referred for coronary angiography despite of excluding T2DM patients or those with fasting PG > 126 mg/dl (7.0 mmol/l) [21-23]. In contrast, in a recent cross-sectional population-based study with over 15,000 non-diabetes patients enrolled, the overall prevalence of AbnGT was approximately 5.6% [24]. In the DECODE study, more than half of diabetic patients had only isolated postchallenge hyperglycemia and three quarters of patients with IGT had normal fasting glucose [5]. In our study, 54% (22 of 41) of patients with IGT and 24% (7 of 29) of patients with T2DM were categorized as having normal fasting glucose. Since several epidemiological studies have indicated that subjects with prediabetic conditions have a raised risk of cardiovascular diseases, early identification of these patients is very important [5,6,25].

### Beyond postchallenge hyperglycemia

In this study, patients with AbnGT were more likely to have CAD or higher numbers of stenosed coronary arteries than those with NGT. However, in comparison between patients with CAD and non-CAD, there were no significant differences among PG-0 to PG-120 during 75 g-OGTT and the PG response curves were almost identical to each other in both NGT and AbnGT groups. Controversial results regarding the correlations of postchallenge hyperglycemia with CAD could be found in previous reports. Kanauchi *et al*. described that postchallenge hyperglycemia was independently associated with the numbers of diseased coronary arteries [26], which was not found in the study reported by Satoh *et al*. [23]. On the other hand, Takezako *et al*. reported that glucose response did not correlate with the severity of coronary atherosclerosis [27]. Accordingly, postchallenge hyperglycemia might not consistently be the only responding factor that causes cardiovascular disease. Instead, it should be considered also as a marker of underlying metabolic abnormalities [9]. These findings from our study highlight the importance of postchallenge proinflammatory and nitrosative stress responses, rather than postchallenge hyperglycemia *per se*, in associations with coronary atherosclerosis in patients without previously diagnosed T2DM.

### Postchallenge proinflammatory and nitrosative responses versus CAD

Most of previous clinical studies reported the baseline levels of inflammatory biomarkers (e.g., C-reactive protein, fibrinogen) and their correlations with postchallenge hyperglycemia or glucose tolerance status after 75 g-OGTT, with or without coronary angiographic data for further discussion about the presence of CAD [28-31]. Esposito *et al*. have shown that consecutive pulses of intravenous glucose could increase circulating TNF-α and IL-6 to a greater extent than during similar and stable glycemic levels, and this effect was more pronounced in subjects with IGT and can be blocked by anti-oxidant [14]. Ceriello *et al*. demonstrated that postprandial hyperglycemia can induce oxidative stress by generation of nitrotyrosine independently and cumulatively in healthy subjects and T2DM patients, but patient with IGT were not included in their study [17,32]. The present study clearly collaborates and extends their findings into patients with IGT, indicating that postchallenge proinflammatory and nitrosative stress responses might be more powerfully correlated to the presence of coronary atherosclerosis than their corresponding levels at baseline.

Although TNF-α, IL-6 and nitrotyrosine can all be elicited time-dependently after 75 g-OGTT, the 2-hr IL-6 levels failed to discriminate the presence of absence of CAD in patients with IGT or T2DM. Nevertheless, it was noteworthy that IL-6 levels at 2-hr were significantly higher in patients with triple-vessel diseases compared with those non-CAD patients regardless of GT status in our study. The potential roles of IL-6 on cardiovascular risk had been postulated in patients with T2DM and unstable CAD [33,34], but the changes of IL-6 had ever been reported poorly correlated to insulin sensitivity in patients with AbnGT [35]. Postchallenge TNF-α and nitrotyrosine levels at 2-hr were significantly higher in patients with CAD and also significantly associated with 2-hr PG. Moreover, in logistic regression analysis after adjusting traditional risk factors and GT status, 2-hr nitrotyrosine levels remained the robust predictor for the presence of CAD in our study. Our findings might not only offer the explanation to the exponential relationship between 2-hr postchallenge glucose levels and the incidence of cardiovascular disease in subjects with IGT from meta-analysis,^2 ^but also indicate that the hyperglycemia-induce atherosclerotic changes were mostly correlated to oxidative stress responses. Meanwhile, the changes of TNF-α might be better and earlier than those of IL-6 in predicating CAD during postchallenge hyperglycemia in patient with AbnGT.

### Association of CAD between NGT and AbnGT: the discrepancy

In our study, significant differences of postchallenge 2-hr nitrotyrosine and TNF-α were identified between CAD and non-CAD patients in AbnGT group, but not in the NGT patients. The possible explanation to this discrepancy might be that both time and level of peak of glucose response curve in the present study tended to be earlier (at 30-60 minutes) and lower in the NGT patients, compared to patients with AbnGT (60-90 minutes) [29]. Similar findings can also be found in the postchallenge response curves of PG or nitrotyrosine after 75 g-OGTT inT2DM and normal control subjects in the other studies [20,24]. The results of early and lower hyperglycemia spike found in NGT patients might render relative insufficient impacts of these postchallenge proinflammatory and nitrosative stress responses to produce sustained effects upon coronary atherosclerotic changes. In addition, it is widely accepted patients with IGT represented more advanced glycemic disorders compared to those NGT patients with insulin resistance but compensatory insulin production [4,33]. Accordingly, postchallenge hyperglycemia-induced proinflammatory cytokines and nitrosative changes on CAD might be been more significantly associated with CAD in patients with AbnGT, but less in patients with NGT.

### Study limitations

This study had several limitations. First, the postchallenge responses of insulin and insulin resistance estimated by homeostasis model assessment (HOMA-IR) were not included in this study. The associations with insulin resistance with the proinflammatory responses and nitrosative stress might need further studies to address. Second, the number of patients in our study was still relatively small. The difference of glucose parameters and IL-6 after 75 g-OGTT might become significant if patients test population increased. However, TNF-α and nitrotyrosine induced by postchallenge hyperglycemia reached statistical significance in our study. Third, the atherosclerotic plaque burden of coronary arteries in our study might be still underestimated by using clinical CAD scoring. Further investigations of coronary atherosclerotic plaque burden evaluated by new imaging modalities, e.g. coronary intravascular ultrasound or mutli-slices computer tomography, to correlate the coronary plaque burden with postchallenge proinflammatory and nitrosative stress responses after acute hyperglycemia might also be warranted.

## Conclusions

High prevalence of AbnGT is demonstrated in patients without previously recognized T2DM referred for coronary angiography. In patients with AbnGT, even under the identical postchallenge hyperglycemia levels, the dynamically postchallenge responses TNF-α and nitrotyrosine are significantly associated with the presence of CAD. Our findings highlight the importance in surveillance of these postchallenge proinflammatory and nitrosative stress biomarkers by 75 g-OGTT for diagnostic and therapeutic assessments of CAD risks in patients without previously recognized T2DM.

## Abbreviations

75 g-OGTT: 75 g oral glucose tolerance test; CAD: Coronary artery disease; IGT: Impaired glucose tolerance; IL-6: Interleukin-6; NGT: Normal glucose tolerance; PG: Plasma glucose; TNF-α: Tumor necrosis factor-alpha; T2DM: Type 2 diabetes mellitus.

## Competing interests

The authors declare that they have no competing interests.

## Authors' contributions

CCS: participating in study design, data analysis, patient enrollment and writing manuscript. LKT, CKH and LMY: participating the study design, patient enrollment and data interpretation. KSF, SHM, LTH, VWC and SSH: participating the patient enrollment and data collection. LWT: designed the study, interpreted the results and revised the manuscript.

All authors read and approved the final manuscript.
